# Effect of Alpha-Lipoic Acid Supplementation on Endothelial Function and Cardiovascular Risk Factors in Overweight/Obese Youths: A Double-Blind, Placebo-Controlled Randomized Trial

**DOI:** 10.3390/nu11020375

**Published:** 2019-02-12

**Authors:** Luciana Tromba, Francesco Massimo Perla, Giovanni Carbotta, Claudio Chiesa, Lucia Pacifico

**Affiliations:** 1 Department of Surgical Sciences, Sapienza University of Rome, 00161 Rome, Italy; luciana.tromba@uniroma1.it (L.T.); giovanni.carbotta@uniroma1.it (G.C.); 2 Department of Pediatrics, Sapienza University of Rome, 00161 Rome, Italy; francescomassimo.perla@uniroma1.it; 3 Institute of Translational Pharmacology, National Research Council, Via del Fosso del Cavaliere, 100, 00133 Rome, Italy; claudio.chiesa@ift.cnr.it

**Keywords:** alpha-lipoic acid, endothelial function, brachial artery flow-mediated dilation, overweight/obese youths

## Abstract

Endothelial dysfunction is recognized as an early sign of systemic atherosclerosis, and it represents a therapeutic target to prevent long-term cardiovascular (CV) consequences. Alpha-lipoic acid (ALA) is a commonly used dietary supplement exerting anti-oxidant and anti-inflammatory effects. We investigated whether a three-month treatment with ALA improves endothelial function, as assessed by flow-mediated dilation (FMD) of the brachial artery, and clinical and metabolic risk factors in overweight/obese youths. We enrolled 67 overweight/obese children, and 22 normal-weight metabolically healthy controls. Overweight/obese youths were randomly allocated in a double-blinded manner to receive ALA (*n* = 34) or placebo (*n* = 33). Of these, 64 (32 ALA, 32 placebo) completed the follow-up. At baseline, in ALA and placebo groups, FMD was similar, but lower as compared with that in controls (*p* = 0.045). At three months, within the ALA and placebo groups, FMD did not change significantly. However, the basal and peak diameter of brachial artery significantly increased after ALA treatment as compared to placebo (*p* = 0.036 and *p* = 0.01, respectively). There were no significant within- and between-group changes for anthropometric and metabolic variables. The results show that ALA supplementation improves vascular tone and may have a beneficial effect on CV health in overweight/obese youths.

## 1. Introduction

With over two billion subjects with overweight/obesity expected worldwide by 2030, atherosclerotic cardiovascular disease (CVD) and premature death will undoubtedly continue to represent relevant public health challenges [1]. Indeed, it is well established that obesity has many adverse effects on cardiometabolic parameters and CV risk factors such as glucose intolerance, type 2 diabetes, atherogenic lipids, blood pressure (BP), and increased levels of inflammation, all of which lead to marked increase in CVD [2]. During the past few decades, obesity shifted toward an onset earlier in life with a dramatic rise in childhood [3]. Importantly, recent reports in children with obesity demonstrated that they may exhibit early signs of cardiovascular dysfunction as a result of excessive adiposity [4], suggesting the problem is not only one of future or long-term CVD risk, but also one requiring immediate attention in childhood to prevent progressive CV damage [5].

Impairment of endothelial function represents a very early step in the development of atherosclerosis, appearing long before clinical symptoms arise, and might qualify as a surrogate endpoint for obesity-associated CVD risk [6]. Under homeostatic conditions, the endothelium maintains normal vascular tone and blood fluidity. However, risk factors including obesity initiate a chronic inflammatory process that is accompanied by reduced release of relaxing signals (nitric oxide (NO)) and anti-thrombotic factors, and by an increase in vasoconstrictor and pro-thrombotic products, resulting in atherosclerotic lesion formation [6]. Thus, endothelial dysfunction is recognized as an early sign of systemic atherosclerosis, and it represents a therapeutic target to prevent long-term CV consequences. 

Emerging evidence suggests that healthy eating habits containing nutritive and non-nutritive compounds may contribute to the improvement of the quality of life by delaying onset and reducing the risk of CVD and, in particular, the development of endothelial dysfunction [7,8]. In this respect, there is great interest in the effects of antioxidant therapy. An antioxidant supplement that generated interest is alpha-lipoic acid (ALA) [9]. ALA (1,2-dithiolane-3-pentanoic acid) is a naturally occurring disulfide compound acting as a cofactor for several mitochondrial enzyme complexes that are involved in energy production. Animal-derived foods (red meat and liver, heart, and kidney) and plant sources (dark green leafy vegetables) [10,11] contain small amounts of ALA, detected in the form of lipoyllysine ranging from 0.55 to 2.36 µg/g dry mass in meats and from 0.16 to 3.15 µg/g dry mass in vegetables [12,13], from which ALA can be released by the combined action of gastric hydrolysis and lipoamidase. However, ALA cannot be detected in the blood of healthy subjects consuming these foods as part of a balanced diet [14]. It is a potent mitochondrial antioxidant agent that acts via multiple mechanisms promoting anti-inflammatory and anti-thrombotic pathways and positively influencing nitric oxide (NO)-mediated vasodilation. ALA was shown to be protective in diseases associated with abnormal oxidative stress and energy metabolism such as diabetes mellitus [15], hyperhomocysteinemia [16], and hypertension [17,18]. Previous studies also revealed that dietary supplementation with ALA was sufficient to improve endothelial function in adult patients with metabolic syndrome [19] and type 2 diabetes [20] and in youths with type 1 diabetes [21]. 

However, to our knowledge, no information is available on whether ALA supplementation has a significant benefit on cardiovascular health in overweight/obese youths. In a double-blind, parallel-group, placebo-controlled randomized trial, we investigated, in children and adolescents with overweight/obesity, the impact of three-month ALA supplementation on endothelial function, as assessed by flow-mediated arterial dilation (FMD), and CVD risk factors. 

## 2. Methods

The study protocol was approved by the local Ethics Committee (Policlinico Umberto I Hospital, Rome, Italy) (Registration trial: 4384/22.03.2017) and written consent was obtained from the next of kin, caretakers, or guardians on behalf of the children enrolled in this study, in accordance with principles of the Helsinki Declaration. 

### 2.1. Study Population

Patients were eligible for the study if they had the following characteristics at enrolment: (1) age between 8 and 16 years; and (2) body mass index (BMI) >85th percentile according to age- and gender-specific percentiles of BMI [22]. Exclusion criteria were pre-existing cardiovascular diseases or inflammatory systemic diseases, history of type 1 or type 2 diabetes, renal disease, smoking and alcohol intake, use of glucocorticoid medications, and previous use of ALA. 

During the same period, normal-weight, metabolically healthy children (age range, 8–16 years) were selected as controls for comparison of FMD at baseline. They were recruited from elementary and middle schools in the Rome area in a pilot program to prevent cardiovascular disease (CVD) in childhood. 

### 2.2. Study Design

The study was a double-blind, parallel-group, placebo-controlled randomized trial performed at the outpatient Clinics of the Department of Pediatrics, Sapienza University of Rome, Italy, between December 2017 and December 2018. Participants were randomly assigned to oral supplementation with ALA (800 mg/dose (Tiobec 800 fast-slow, Laborest, Italy)) or placebo containing the same excipients (i.e., maltodextrins, thickening agents (acacia gum, xanthan gum), coating agents (aroma), coloring agents (beta-carotene), and sweeteners (sucralose)) as the ALA formulation, both in a sachet formulation (granules) once daily for 12 weeks. A randomization list (in a 1:1 ratio to treatment with ALA or placebo) was generated by an independent statistician who was blinded to participants’ clinical data. All participants and research staff were blind to the group assignment. Compliance to treatment was encouraged by weekly phone calls and text messages and monitored by direct interview at every monthly visit. Adverse events were defined as those related to or caused by the treatments under study. At each visit, parents were specifically asked about adverse events, and the second author (F.M.P.) checked for any association between the adverse events and morbidity. 

A balanced low-calorie diet was prescribed to all patients with a recommendation to engage in a moderate daily exercise program (60 min/day at least five days a week), and to reduce sedentary activities. Specifically, diet was hypocaloric (25–30 calories/kg/day), consisting of carbohydrate (50–60%), protein (15–20%), and fat (23–30%), with a composition of two-thirds unsaturated and one-third saturated. 

To evaluate adherence to lifestyle recommendations, one-week weighted dietary and physical activity records were used. 

### 2.3. Ultrasound Measures of the Diameter and Flow-Mediated Dilation of the Brachial Artery 

Assessment of FMD was performed by a single experienced FMD reader (L.T.) according to the guidelines of the International Brachial Artery Reactivity Task Force [23]. The brachial artery was scanned above the antecubital fossa of the right arm using high-resolution vascular ultrasonography (Mylab 70 XVision Gold, 7-15-MHz linear-array transducer, Esaote, Genova, Italy). Longitudinal, electrocardiogram-gated, end-diastolic images were acquired of the brachial arterial diameter over a 1- to 2-cm segment, and computer-assisted edge detection brachial analysis software was used to measure the brachial artery diameters. Brachial artery diameters were measured prior to, and then 45 and 70 seconds after five minutes of reduced blood flow (induced by inflation of a standard sphygmomanometer cuff, placed at the mid–upper arm, to at least 50 mm Hg above resting systolic blood pressure (BP)). FMD was assessed as the percentage change from baseline to maximal diameter of the brachial artery with reactive hyperemia (FMD = peak diameter − baseline diameter/baseline diameter). The average of three measurements at each time point was used to derive the maximum FMD. FMD was measured a second time by the same physician in 15 randomly selected healthy study children, producing a considerably high intra-correlation coefficient (0.98). 

### 2.4. Clinical and Laboratory Data

All participants underwent physical examination including weight measurement, standing height, BMI, waist circumference (WC), determination of pubertal status, and systolic and diastolic BP, as reported in detail previously [24]. The degree of obesity was quantified using Cole’s least-mean-squares method, which normalizes the skewed distribution of BMI and expresses BMI as a standard deviation score (SDS) [22]. 

Blood samples were taken from each subject, after an overnight fast, for estimation of glucose, insulin, total cholesterol, high-density lipoprotein cholesterol (HDL-C), low-density lipoprotein cholesterol (LDL-C), triglycerides, alanine aminotransferase (ALT), and high-sensitivity C reactive protein (HSCRP). Estimates of insulin sensitivity were calculated using the homeostasis model assessment of insulin resistance (HOMA-IR) (fasting insulin (μU/mL) × fasting glucose (mmol/L)/22.5). All analyses were conducted using COBAS 6000 (Roche Diagnostics). Insulin concentrations were measured on a cobas e 601 module (Electrochemiluminescence Technology, Roche Diagnostics), while the remaining analytes were measured on a cobas e 501 clinical chemistry module (Photometric Technology). 

### 2.5. Statistical Analysis

Statistical analyses were performed using the SPSS package (version 25.0), SPSS Inc., Chicago, IL, USA. The primary endpoint used for the sample size determination was the change in percentage FMD. The sample size was calculated by a power analysis using our previous data [25] with the following assumptions: a type 1 error of 0.05 (two-tailed), 80% power, a mean increase in FMD of 10% in the ALA group, a mean increase of 0% in the placebo group, and a standard deviation of 10% in each group. A minimum of 46 subjects (after a potential 10% drop out), 23 per group, would provide 80% power to detect a difference in the percentage change of FMD. Secondary outcomes were the changes in BMI-SDS score, WC, BP, lipids, and HOMA-IR. Data are reported as means and standard deviations for normally distributed variables, or as medians and interquartile ranges for non-normally distributed variables. Baseline differences between the three study groups were evaluated by ANOVA or Kruskal–Wallis test, as appropriate. Proportions were compared using the chi-square test. Changes from baseline to three months were compared using a paired *t*-test or Wilcoxon’s rank sum test within each group, and using a *t*-test or Mann–Whitney *U*-test between groups. A *p*-value <0.05 was considered significant. 

## 3. Results

### 3.1. Patient Characteristics

**Figure 1:**
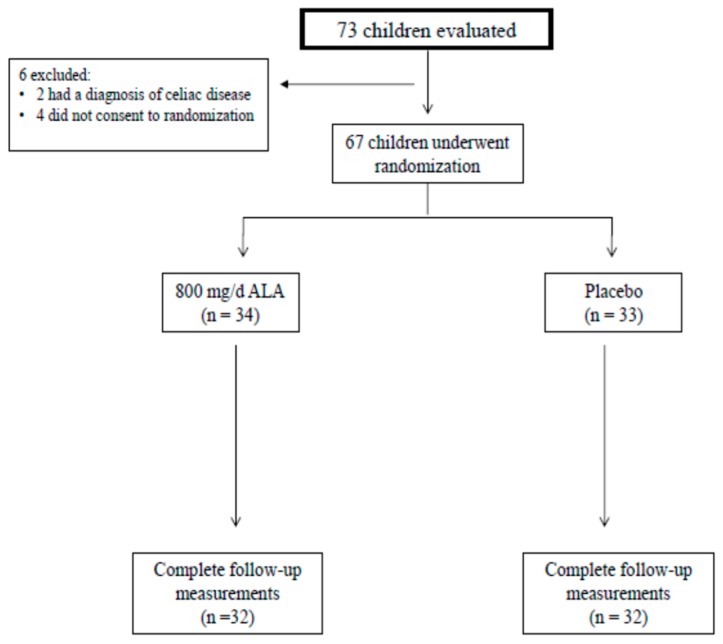
Flowchart of the study.

We evaluated 73 overweight/obese children at our outpatient clinic. While two subjects were excluded because of a diagnosis of celiac disease, four did not give consent to randomization. Thus, a total of 67 children were randomized to treatment with ALA (*n* = 34) or placebo (*n* = 33) for three months (Figure 1). Of these, two patients from the ALA and one patient from the placebo groups were excluded from the efficacy evaluable set. The reason was low compliance. There were no adverse events in either group. The final analysis was performed with a total of 64 participants who received ALA (*n* = 32) or placebo (*n* = 32). Controls included 22 healthy children and adolescents with BMI appropriate for age and gender, and normal values for biochemical analyses.

Characteristics of study subjects at baseline are presented in Table 1 and Table 2. As expected, anthropometric and metabolic data were significantly different between obese patients and metabolically healthy subjects, with the exception of glucose levels (Table 1). Also, the basal and peak diameter of the brachial artery, and the FMD were different between overweight/obese children and healthy controls. In fact, at baseline, FMD in ALA- and placebo-treated groups were 12.5% (interquartile range, 6.8–23.0) and 12.5% (4.0–20.0), respectively, which was lower than that in controls (24.0% (17.8–28.0); *p* = 0.045) (Table 2). In contrast, between the two randomized groups (Table 1 and Table 2), there were no significant differences in pre-treatment clinical, metabolic, and vascular characteristics. 

In the entire cohort, univariate analysis showed a significant correlation between baseline FMD and BMI-SDS (*r* = −0.310; *p* = 0.002), WC (*r* = −0.256; *p* = 0.012), systolic BP (*r* = −0.336; *p* = 0.001), diastolic BP (*r* = −0.290; *p* = 0.004), LDL-C (*r* = −0.272; *p* = 0.003), insulin (*r* = −0.313; *p* = 0.003), and HSCRP (*r* = −0.271; *p* = 0.008). In multiple regression analysis, FMD was significantly associated with age, BMI-SDS, and LDL-C concentrations. In the group of overweight/obese youth, after adjustment for age and gender, FMD was significantly associated with LDL-C levels.

### 3.2. Effects of ALA on FMD (Main Outcome) and Clinical and Metabolic Variables 

After the three-month treatment, there were no significant differences within each group for BMI-SDS, WC, waist-to-height ratio, and BP. Among metabolic variables, there was only a mild but not significant decrease in LDL-C cholesterol after ALA treatment (*p* = 0.057) (Table 3). 

**Figure 2:**
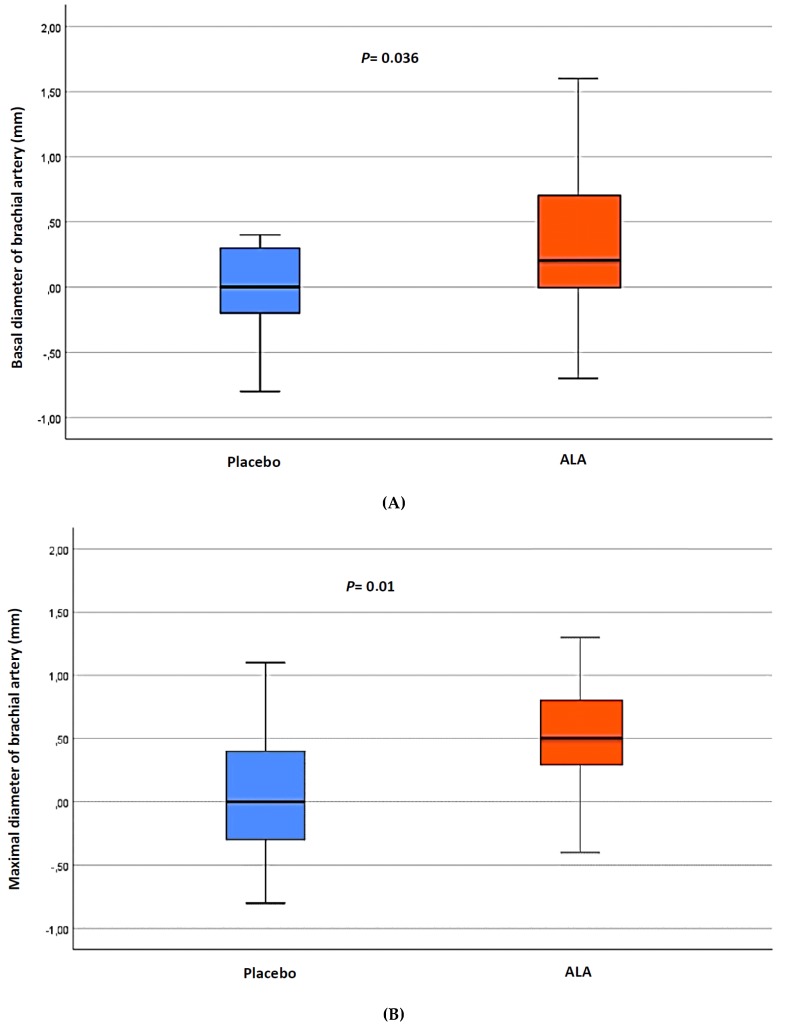
Change in basal (**A**) and maximal (**B**) brachial artery diameter at three-month follow-up.

Table 4 shows the results of basal brachial artery diameter, maximal brachial artery diameter, and FMD before and after the three-month treatment. FMD (expressed as percentage change from basal diameter) did not change significantly within the ALA (12.5% (interquartile range, 6.8–23.0) to 16.7% (interquartile range, 10.9–23.9); *p* = 0.28) and within the placebo (12.5% (4.0–20.0) to 12.5% (5.5–18.2); *p* = 0.24) groups. However, a marked peripheral vasodilation was observed following ALA treatment versus placebo. In this respect, while, within the ALA group, the increase in the brachial artery diameter was evident before reactive hyperemia (basal, 2.8 (interquartile range, 2.6–3.1) mm to 3.3 (3.1–3.6) mm; *p* = 0.011), as well as at the peak dilation following cuff release (maximal, 3.4 (3.1–3.6) mm to 3.9 (3.7–4.2) mm; *p* = 0.001), within the placebo group, both basal and maximal brachial artery diameters remained similar after the three-month follow-up. There were statistically significant differences in the percentage change in the basal (*p* = 0.036) and maximal (*p* = 0.01) diameters between the two groups (Table 4, Figure 2). 

## 4. Discussion

We performed a double-blind, parallel-group, placebo-controlled randomized trial to study the effects of treatment with ALA at a dose of 800 mg/day for three months on endothelial function in overweight/obese youths. In addition, we assessed the effect of ALA on markers of cardiovascular risk including BP, lipid profile, insulin resistance, and HSCRP. Our study demonstrated that treatment with ALA was associated with significant increases in basal and peak arterial diameters, reaching values observed in the group of normal-weight healthy subjects. However, more targeted measures of vascular endothelial function such as FMD remained unchanged. It is possible that FMD change in our small population could have been attenuated by the increment in baseline diameter, which might have masked the percent change in the diameter after stimulus. To our knowledge, this is the first study to examine the effects of an antioxidant such as ALA on arterial endothelial function in overweight/obese youths. The observed vascular changes reflect vasodilation and reduced arterial tone. Similar findings were observed in a study in which adult participants with coronary artery disease received combined alpha-lipoic acid/acetyl-l-carnitine for eight weeks [26]. In this regard, the combined treatment increased basal brachial artery diameter by 2.3%, consistent with reduced arterial tone; in contrast, no effect of treatment on the dilator responses to nitroglycerin or ischemia was observed. 

The mechanisms for the vasodilator effect of ALA treatment remain undefined in our study. We cannot rule out that the sustained improvement in arterial tone might have been caused by an enhanced endothelial release of NO, although FMD of the brachial artery remained unchanged. As previously emphasized in other trials performed in a different clinical setting [27], we reiterate the importance of reporting arterial diameters in addition to FMD in future publications. 

Alpha-lipoic acid and its reduced form, dihydrolipoate, are potent antioxidants. They are amphiphilic and widely distributed in both the cell membrane and cytosol. Alpha-lipoic acid was used in Germany for patients with neuropathy for over 30 years, and is considered to be safe and efficacious for treatment of diabetic symptomatic polyneuropathy [28]. In recent years, it was suggested that oxidative stress contributes to endothelial dysfunction and there is convergent evidence from clinical trials, animal models, and in vitro studies that ALA may enhance endothelium-dependent vasodilation primarily by increasing NO production [29]. In 2001, Heitzer et al. demonstrated that ALA in a therapeutic dose improves endothelial dysfunction in diabetic patients by increasing NO-mediated vasodilation [30]. A subsequent study showed that ALA can improve endothelial dysfunction induced by acute hyperglycemia during oral glucose tolerance test in impaired glucose tolerance (IGT) [31]. Yet, in subjects with IGT who showed impaired FMD, treatment with ALA (600 mg via intravenous infusion once a day for three weeks) was associated with improvement of endothelial function through a decrease in oxygen-derived free radicals [32]. The authors measured the serum levels of nitrite/nitrate, metabolites, and markers for production of NO, as well the plasma lipid peroxide content using thiobarbituric acid reactive substances (TBARS), a marker of oxygen-derived free radicals. Indeed, FMD decreased in association with an increase in plasma levels of TBARS, while levels of nitrite/nitrate did not change, suggesting a quenching of NO rather than a decreased production/release of NO. 

Previous studies observed improvements in components of metabolic syndrome with the supplementation of ALA. In particular, three recent systematic reviews and meta-analyses of randomized controlled clinical trials summarized the effects of ALA supplementation on anthropometric (body weight, BMI, and WC), metabolic (fasting glucose, insulin, HOMA, hemoglobin A1C, triglycerides, total cholesterol, LDL-C, and HDL-C), and inflammatory (CRP) indices among adult subjects, respectively [33,34,35]. The first systematic review and meta-analysis revealed that ALA supplementation slightly but significantly decreased body weight and BMI. However, it did not decrease WC compared with the placebo group [33]. The second one showed that ALA administration may lead to improvement in glucose homeostasis parameters and lipid profiles except HDL-C [34]. The third systematic review and meta-analysis showed that ALA supplementation could significantly decrease CRP level only when the baseline concentration of this inflammatory marker was elevated (>3 mg/L) [35]. In the present study involving overweight/obese youths, we found no effect of ALA supplementation on anthropometric indices, as well as on glucose homeostasis parameters, HSCRP levels, and lipid profiles, although a trend was observed for LDL-C levels. The discrepant results between our study and previous ones could be attributable to several reasons such as study population (including demographic and clinical characteristics, lifestyle factors, underlying diseases, and type and onset of complications), type of intervention (ALA supplements versus ALA plus other nutrients), route (oral versus parenteral), dosage and duration of ALA administration, and sample size of the study. 

The main strengths of our study are the parallel-group, double-blind, randomized controlled trial design; the careful clinical characterization of the participants with their CV risk factors; and the meticulous conduct of the vascular procedures. Despite these strengths, our study has limitations. Firstly, the small sample size may limit the interpretation of the results, but could provide indications for further research. Secondly, LA supplement was given for only three months. However, a three-month duration of treatment is frequently applied in clinical trials with dietetic supplements and is considered sufficient to achieve therapeutic results and maintain good compliance. Thirdly, although vascular effects of ALA are attributed to their antioxidant effects, we did not assess the measures of oxidative stress in the present study. This limits our ability to draw conclusions about the mechanism responsible for vasodilation. 

## 5. Conclusions

In conclusion, in the present placebo-controlled, randomized trial, we observed substantial reductions in vascular constriction in the brachial artery in children and adolescents with overweight/obesity. Additional prospective studies with a larger sample size are necessary to reveal the efficacy of this nutraceutical supplement as part of a multifaceted approach in the primary prevention of atherosclerosis in unhealthy overweight and obese children and adolescents.

## Figures and Tables

**Table 1:** Characteristics of study groups at baseline.

Variables	Controls (*n* = 22)	Obese		*p* *
		Placebo (*n* = 32)	ALA (*n* = 32)	
Age, years	11.2 ± 2.8	11.1 ± 2.1	11.5 ± 1.9	0.64
Male gender, *n* (%)	13 (59.1)	20 (62.5)	16 (50.0)	0.58
Prepubertal status, *n* (%)	4 (18.2)	2 (6.25)	2 (6.25)	0.25
Weight, kg	38.8 ± 14.8	60.8 ± 15.2	64.2 ± 14.1	<0.0001
BMI	17.7 ± 2.3	26.0 ± 2.8	27.4 ± 3.5	<0.0001
BMI-SDS	−0.01 ± 0.68	1.94 ± 0.33	1.94 ± 0.46	<0.0001
Waist circumference, cm	65.2 ± 11.2	86.0 ± 10.7	85.0 ± 14.6	<0.0001
Waist-to-height ratio	0.45 ± 0.08	0.57 ± 0.09	0.56 ± 0.06	<0.0001
Systolic BP, mmHg	98.2 ± 12.2	116.8 ± 7.1	117.0 ± 9.0	<0.0001
Diastolic BP, mmHg	70.0 ± 7.1	70.5 ± 7.1	69.4 ± 7.9	<0.0001
ALT, U/L	13.5 (12.0–16.5)	16.0 (14.0–38.0)	18.0 (14.0–23.0)	0.01
TC, mg/dL	151 (132–162)	162 (133–182)	172 (147–192)	0.012
HDL-C, mg/dL	58 (50–64)	48 (40–53)	49 (46–60)	0.005
LDL-C, mg/dL	75 (63–89)	90 (68–114)	95 (79–123)	0.005
TG, mg/dL	50 (46–70)	93 (77–118)	95 (72–129)	<0.0001
Glucose, mg/dL	85.0 ± 7.0	82.4 ± 7.7	83.8 ± 6.0	0.35
Insulin, μU/mL	7.2 (5.1–9.7)	15.3 (11.0–22.3)	16.2 (11.9–24.9)	<0.0001
HOMA-IR	1.34 (1.0–1.9)	3.01 (2.26–5.19)	3.37 (2.27–5.0)	<0.0001
2-hour glucose, mg/dL	ND	94.5 ± 15.7	94.7 ± 10.9	0.61
2-hour insulin, µU/mL	ND	41.7 (27.6–92.7)	45.2 (30.8–76.4)	0.40
HSCRP, μg/L	350 (300–725)	1400 (925–2675)	1250 (925–1675)	<0.0001

Results are expressed as *n* (%), mean (SD), or median (IQR). * *p*-values indicate comparison among the three groups; post hoc analysis showed no significant differences in baseline clinical and metabolic variables between placebo and ALA groups. BMI, body mass index; BMI-SDS, body mass index standard deviation score; BP, blood pressure; ALT, alanine aminotransferase; TC, total cholesterol; HDL-C, high-density lipoprotein cholesterol; LDL-C, low-density lipoprotein cholesterol; TG, triglycerides; HOMA-IR, homeostasis model assessment of insulin resistance; ND, not done; HSCRP, high-sensitivity C reactive protein.

**Table 2:** Brachial artery measurements of study groups at baseline.

Variables	Controls	Obese		*p* *
		Placebo	ALA	
Basal brachial artery diameter, mm	3.35 (3.1–3.7)	2.9 (2.55–3.2)	2.8 (2.65–3.15)	0.0001
Peak brachial artery diameter, mm	4.0 (3.8–4.7)	3.3 (3.1–3.6)	3.4 (3.1–3.65)	0.0001
FMD, %	24.0% (17.8–28.0)	12.5 (4.0–20.0)	12.5 (6.8–23.0)	0.045

Results are expressed as median (interquartile range). * *p*-values indicate comparison among the three groups. FMD, flow-mediated dilation.

**Table 3:** Anthropometric and cardiovascular risk factors before and after intervention with placebo or ALA in obese youths.

Variables		Placebo			ALA		
	Baseline	End of Study	*p* *	Baseline	End of Study	*p* *	p †
Weight, kg	60.8 ± 15.2	61.2 ± 13.6	0.32	64.2 ± 14.1	64.1 ± 14.6	0.35	0.94
BMI-SDS	1.94 ± 0.33	1.89 ± 0.35	0.16	1.94 ± 0.46	1.89 ± 0.45	0.46	0.97
WC, cm	86.0 ± 10.7	86.2 ± 9.3	0.65	85.0 ± 14.6	86.7 ± 9.9	0.38	0.24
Waist-to-height ratio	0.57 ± 0.09	0.56 ± 0.05	0.99	0.56 ± 0.09	0.56 ± 0.06	0.69	0.25
Systolic BP, (mm Hg)	116.8 ± 7.1	114.5 ± 8.5	0.31	117.0 ± 9.0	117.7 ± 10.4	0.78	0.74
Diastolic BP, (mm Hg)	70.5 ± 7.1	69.0 ± 7.7	0.39	69.4 ± 7.9	69.1 ± 12.6	0.95	0.25
ALT, U/L	16 (14–38)	16 (12–23)	0.30	19 (14–23)	19 (14–24)	0.68	0.25
TC, mg/dL	162 (133–182)	165 (133–179)	0.44	172 (147–192)	169 (136–188)	0.09	0.64
HDL-C, mg/dL	48 (40–53)	46 (38–52)	0.15	49 (46–60)	51 (44–56)	0.13	0.12
LDL-C, mg/dL	90 (68–114)	91 (72–112)	0.17	95 (79–123)	93 (74–114)	0.057	0.62
TG, mg/dL	93 (77–118)	90 (68–115)	0.13	95 (72–129)	90 (76–113)	0.15	0.11
Glucose, mg/dL	82.4 ± 7.7	80.9 ± 17.7	0.51	83.8 ± 6.0	83.1 ± 16.8	0.89	0.15
Insulin, μU/mL	15.3 (11.0–22.3)	16.3 (10.9–20.0)	0.35	16.2 (11.9–24.9)	17.0 (12.1–23.9)	0.68	0.90
HOMA-IR	3.01 (2.26–5.19)	3.18 (2.19–5.19)	0.44	3.37 (2.27–5.0)	3.41 (2.0–4.94)	0.87	0.50
HSCRP, μg/L	1400 (925–2675)	1100 (500–3200)	0.99	1250 (925–1675)	1100 (800–2250)	0.18	0.56

Results are expressed as *n* (%), mean (SD), or median (IQR). * *p*-values indicate comparison within each group; **†** *p*-values indicate comparison of the changes of each variable between the two groups. BMI, body mass index; BMI-SDS, body mass index-standard deviation score; WC, waist circumference; BP, blood pressure; ALT, alanine aminotransferase; TC, total cholesterol; HDL-C, high-density lipoprotein cholesterol; LDL-C, low-density lipoprotein cholesterol; TG, triglycerides; HOMA-IR, homeostasis model assessment of insulin resistance; HSCRP, high-sensitivity C reactive protein.

**Table 4:** Brachial artery measurements after intervention with placebo or ALA in obese youths.

Variables		Placebo			ALA		
	Baseline	End of Study	*p* *	Baseline	End of Study	*p* *	*p* †
Basal brachial artery diameter, mm	2.9 (2.5–3.2)	2.9 (2.8–3.2)	0.062	2.8 (2.6–3.1)	3.3 (3.1–3.6)	0.011	0.036
Peak brachial artery diameter, mm	3.3 (3.1–3.6)	3.4 (3.1–3.6)	0.29	3.4 (3.1–3.6)	3.9 (3.7–4.2)	0.001	0.01
FMD, %	12.5 (4.0–20.0)	12.5 (5.5–18.2)	0.24	12.5 (6.8–23.0)	16.7 (10.9–23.9)	0.28	0.13

Results are expressed as median (interquartile range). * *p*-values indicate comparison within groups; **†** *p*-values indicate comparison of the changes of each variable between the 2 groups. FMD, flow-mediated dilation.
